# The Dynamics of Concussion: Mapping Pathophysiology, Persistence, and Recovery With Causal-Loop Diagramming

**DOI:** 10.3389/fneur.2018.00203

**Published:** 2018-04-04

**Authors:** Erin S. Kenzie, Elle L. Parks, Erin D. Bigler, David W. Wright, Miranda M. Lim, James C. Chesnutt, Gregory W. J. Hawryluk, Wayne Gordon, Wayne Wakeland

**Affiliations:** ^1^Systems Science Program, Portland State University, Portland, OR, United States; ^2^Department of Psychology and Neuroscience Center, Brigham Young University, Provo, UT, United States; ^3^Department of Emergency Medicine, Emory University School of Medicine, Atlanta, GA, United States; ^4^Sleep Disorders Clinic, Division of Hospital and Specialty Medicine, Research Service, VA Portland Health Care System, Portland, OR, United States; ^5^Departments of Neurology, Medicine, and Behavioral Neuroscience, Oregon Institute of Occupational Health Sciences, Oregon Health & Science University, Portland, OR, United States; ^6^TBI/Concussion Program, Orthopedics & Rehabilitation, Neurology and Family Medicine, Oregon Health & Science University, Portland, OR, United States; ^7^Department of Neurosurgery, University of Utah, Salt Lake City, UT, United States; ^8^Department of Rehabilitation Medicine, Icahn School of Medicine at Mount Sinai, New York, NY, United States

**Keywords:** concussion, traumatic brain injury, systems science, complexity, recovery, causal-loop diagram, models of injury, systems medicine

## Abstract

Despite increasing public awareness and a growing body of literature on the subject of concussion, or mild traumatic brain injury, an urgent need still exists for reliable diagnostic measures, clinical care guidelines, and effective treatments for the condition. Complexity and heterogeneity complicate research efforts and indicate the need for innovative approaches to synthesize current knowledge in order to improve clinical outcomes. Methods from the interdisciplinary field of systems science, including models of complex systems, have been increasingly applied to biomedical applications and show promise for generating insight for traumatic brain injury. The current study uses causal-loop diagramming to visualize relationships between factors influencing the pathophysiology and recovery trajectories of concussive injury, including persistence of symptoms and deficits. The primary output is a series of preliminary systems maps detailing feedback loops, intrinsic dynamics, exogenous drivers, and hubs across several scales, from micro-level cellular processes to social influences. Key system features, such as the role of specific restorative feedback processes and cross-scale connections, are examined and discussed in the context of recovery trajectories. This systems approach integrates research findings across disciplines and allows components to be considered in relation to larger system influences, which enables the identification of research gaps, supports classification efforts, and provides a framework for interdisciplinary collaboration and communication—all strides that would benefit diagnosis, prognosis, and treatment in the clinic.

## Introduction

Concussion, also known as mild traumatic brain injury (mTBI),^1^ is a significant public health issue responsible for a variety of cognitive, emotional, and somatic symptoms and deficits (3). It is unclear why some individuals appear to recover relatively quickly while others suffer prolonged symptoms and impairments (4–7). Robust clinical means of diagnosis, prognosis, and treatment are also lacking (8–11). Research is hindered by an inadequate classification system for traumatic brain injury (TBI) (12), “poor” study quality (13, 14), disagreement about appropriate inclusion and exclusion criteria for concussion (8, 15), and an incomplete understanding of underlying pathophysiology (16–18). The heterogeneity and complexity seen in concussion further complicate research, particularly efforts to individualize treatment (19–22).

The study and clinical care of concussion spans many disciplines. Integrating research findings into a common framework would yield benefits for interdisciplinary communication, but such integration faces significant challenges. Diverse specialties use different definitions of concussion, models of injury, and measures of progress toward recovery (23). Translation of findings from animal models is particularly challenging when studying subtle changes in human consciousness (20). Initial efforts to improve data agreement, such as the Common Data Elements^2^ and Federal Interagency Traumatic Brain Injury Research,^3^ have made important strides toward interdisciplinarity, but are constrained by the lack of a shared explanatory model upon which to base a new system of classification (24). Recent biopsychosocial models combining multiple variables of concussion recovery have been proposed (25–27), but their linear formulation and limited scope fall short of capturing the complex, interdependent network of factors and nonlinear recovery trajectories often seen with concussion. A recent article published by researchers associated with the International Initiative for Traumatic Brain Injury Research (22) highlights the importance of “multidimensional approaches” and prognostic models for improving clinical outcomes.

Systems science is an interdisciplinary field of study offering diverse methods and theories for the study of complex systems. A key idea underlying the systems approach is an emphasis on the interconnections between system components and how mutual causality impacts overall system behavior or performance. These methods can be used to better understand complex public health issues by providing ways of visualizing and analyzing systems of interest (28, 29). By synthesizing information from diverse stakeholders and fields, they can serve as an organizing framework for current knowledge and support the development of a shared understanding of complex phenomena (30). In recent years, this approach has been increasingly applied to biomedical and public health issues such as obesity (31), drug diversion and abuse (32), and depression (33).

Previous research conducted by several of the current authors examined the state of knowledge about concussion through a systems lens and identified key variables relevant to recovery across multiple scales (19). The current research builds upon that work by producing a detailed systems model of concussion pathophysiology and persistence or recovery of symptoms, with a focus on the feedback relationships underlying nonlinear system behavior. This paper introduces key systems concepts to the TBI community and provides an opportunity to examine whether useful insight can be generated using a novel systems mapping approach.

Treating concussion injury and recovery as a complex, interdependent system of physiological, experiential, and social variables influenced by a heterogeneous array of personal and injury characteristics is a significant shift from conventional approaches to medical research in which variability is minimized in order to identify correlations between a small number of variables. Working in conjunction with traditional reductionist research that can identify individual relationships from controlled experiments and newer big-data efforts that can identify patterns in large sets of data, systems science methods enable a big-picture perspective that can inform research. Because clinical and research disciplines in medicine are highly specialized, opportunities to develop holistic perspectives are rare. Systems science methods are particularly well suited to a key challenge in brain injury research: understanding mechanisms underlying heterogeneous recovery trajectories, in order to improve clinical prediction models and classification of patients at various time points in recovery. By analyzing how variables interrelate to enable symptom resolution or persistence, the complex nature of concussion can be better understood.

## Materials and Methods

To address current questions about heterogeneity, classification, and lack of a shared explanatory framework in concussion, the Brain Trauma Foundation convened an interdisciplinary panel of researchers and clinicians in the field of TBI as part of the Brain Trauma Evidence-based Consortium (B-TEC). Model development was led by a core methods team with expertise in systems science and neuroscience. The model was developed iteratively, with a high degree of involvement from TBI researchers and clinicians. Several researchers provided close support throughout the multi-year process and are included as coauthors on the current paper, while a series of in-person meetings allowed input from B-TEC investigators. Presentation of in-progress models at several conferences allowed for feedback from broader communities of TBI researchers and systems modelers. Rounds of literature review and individual interviews with 26 experts (Table S1 in Supplementary Material) further guided model development. Interviews were semi-structured, conducted by members of the core methods team, and typically lasted 60–90 min. Transcriptions of the interviews were coded and analyzed for relevant content. Model revisions were made in an iterative fashion as new information was gathered.

Review of the literature was informed by guidance from experts and needs that were identified during the process of model building. The review of published, peer-reviewed literature was extensive, though not systematic; due to known data quality issues with the existing literature (34), the choice was made to take an inclusive approach. To build a comprehensive model using only studies meeting the highest methodological standards would have been impossible since very few such studies exist (34). Furthermore, including a wide variety of types of peer-reviewed research allows for a richer, albeit more speculative, view of the system to emerge.

In an earlier phase of the project, the team outlined the application of a systems approach to concussion and developed systems diagrams outlining key variables across four emergent, interconnected scales: cellular, network, experiential, and social (19). For the current phase, the team further expanded and specified these models as causal-loop diagrams (CLDs) to describe interconnections between system variables in more detail. CLDs use a simple notation to map hypothesized causal relationships between variables in aggregate quantities (35). This method is particularly useful for revealing feedback loops, which often generate nonlinear dynamics in complex systems.

Unlike statistical models extrapolated or imputed from correlations in relevant data, systems models are often built in a top-down fashion based on causal hypotheses of how the systems are thought to operate. Such a model serves as a reflection of the knowledge and assumptions held by a person or group—a shared *mental model* in the systems literature (36). When empirical or theoretical knowledge about the target system is incomplete, a systems model can identify where to focus attention for future empirical investigation.

The goal of building this model is to depict relationships between key variables influencing concussion pathophysiology and symptomatology across multiple scales, with a focus on identifying endogenous feedback mechanisms that shape recovery trajectories. While the heterogeneity of concussion implies that one static model cannot be universally applicable across all patients, an effort was made to include variables and relationships common to many cases. The team identified primary system components from interview data and review of the literature, then, modeled them at a scale determined by their connections to other system components. This resulted in a model describing pathophysiology and recovery at several scales. Priority was given to articulating how and why post-concussive symptoms might persist over time.

Due to evidence indicating distinct pathophysiological mechanisms underlying blast injury (16, 37–39), the decision was made to focus exclusively on concussion (or mTBI) caused by blunt impact or accelerative/decelerative forces. Also excluded were penetrating injuries, as they are generally associated with more severe TBI, including more focal deficits related to the specific location of injury.

Documentation of supporting evidence was done alongside model building, and was ultimately compiled in the Evidence Table (Table S2 in Supplementary Material).

After building the model, the core team conducted loop analysis to identify feedback loops and key connections. Supplementary diagrams were created to communicate noteworthy patterns in loop structure.

## Results: CLD

**Figure 1:**
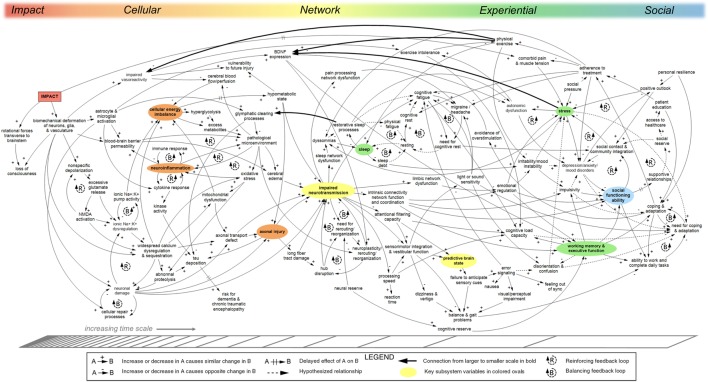
Causal-loop diagram (CLD) of concussion pathophysiology, persistence, and recovery. The model shows causal relationships between factors influencing recovery from impact concussion. Reinforcing feedback loops characterized by exponential growth or decline are indicated with R-loop symbols; balancing feedback loops for repair, replenishing, or homeostatic processes that strive to move the systems toward a set point are indicated with B-loop symbols. Hash marks across an arrow indicate a significant delay. Approximate organization across biological scales is shown on the top axis; change in time scale is indicated on the bottom axis. Key indicator variables representative of subsections of the model are shown in colors corresponding to the top axis. Solid arrows indicate relationships identified in the literature. Relationships not supported directly by published literature are shown with dotted arrows. “Downscale” connections linking variables at larger scales to variables at smaller scales are indicated in bold. The model was developed qualitatively based on iterative review of relevant literature, expert interviews, and expert review. A web-based interactive version of this diagram can be found at www.dynamicsofconcussion.com. Supporting documentation can be found in the Evidence Table (Table S2 in Supplementary Material). Diagram rendered in MapSys (Simtegra Version 4.0).

The CLD shown in Figure 1 provides an interdisciplinary, multi-scale depiction of key feedback dynamics related to concussion, including persistent post-concussive syndrome. The model includes injury to the brain from blunt impact or accelerative/decelerative forces, but is not intended to describe blast injury. The diagram shows a system that is highly complex, with many feedback loops and connections between subsystems operating at different biological and time scales. The model is intended as a preliminary demonstration of this method and only captures a portion of the immense complexity of concussion recovery. An interactive, web-based reproduction of Figure 1 was generated using Kumu (40) and can be found at www.dynamicsofconcussion.com.

The complex, highly interconnected nature of the system of factors relevant to concussion pathophysiology and recovery is readily apparent in Figure 1. The large number of incoming and outgoing arrows connected to many of the variables indicates a system in which causality is shared across a diverse set of factors. After providing an orienting narrative of the systems model, specific aspects such as feedback dynamics, drivers, hubs, and boundaries will be presented.

### Model Narrative

From left to right, Figure 1 shows variables at increasing time scales and at several scales of biological organization: cellular, network, experiential, and social. This section describes in narrative form the relationships seen in Figure 1. Note that neither the model nor the corresponding narrative is exhaustive. Rather, they reference only system components identified during the course of our research.

#### Cellular

Immediately following impact to the brain (from either a blunt force or rapid acceleration/deceleration of the head), biomechanical stretch/strain effects from deformation may alter the cytoskeletal structure of neurons, glia, vasculature, and the structural extracellular matrix (41). This disruption initiates a variety of acute neuroanatomic, neurotransmitter, neurometabolic, inflammatory, and vascular processes at the cellular level (42). Physical alteration from transient disruption of brainstem–cortical connections may include loss of consciousness if rotational forces transverse to the brainstem are present (43, 44). Excessive glutamate and ionic flux contribute to an energy crisis, which can result in a prolonged hypometabolic state following injury (42). Calcium dysregulation persists longer than other ionic disruptions and can cause mitochondrial dysfunction and oxidative stress, and exacerbate the cellular energy imbalance (42). The byproducts of various cellular processes can create a pathological microenvironment, which puts further stress on cellular networks (45). Physical damage to astrocyte and microglial cells, along with their activation in response to injury, can cause permeability in the blood–brain barrier whereby peripheral leukocytes and other compounds contribute to a pathological microenvironment causing neuroinflammation (46, 47). If neurometabolism is disrupted for an extended period of time, mechanisms of neuronal damage are initiated due to calcium sequestration and abnormal proteolysis (42), as well as axonal injury, *via* extended hypometabolism and various defects in axonal transport (48–52).

#### Network

Disruption at the cellular level directly impairs neurotransmission, which causes both generalized as well as focal functional impairment in any number of neural networks, depending on the location of impairment, the cell types impacted, and the large-scale population dynamics of local and global neuronal ensembles (53–55). Impaired neurotransmission serves as a key hub in our model and a point of emergence between cellular-scale processes and neural networks (56, 57). Using *impaired neurotransmission* as an aggregate variable excludes the spatially localized information gleaned from neuroimaging and histological studies. While these approaches are crucial to better understanding injury heterogeneity, the current project focuses instead on the causes and effects of neurotransmission impairment in order to illustrate system-level nonlinear feedback dynamics responsible for symptom persistence and recovery across heterogeneous concussive injuries.

Impaired neurotransmission is exacerbated by damage to long fiber tracts and hub disruption, and is ameliorated by processes of neuroplasticity, rerouting, and reorganization (58). The manner in which network dysfunction is repaired or manifests into symptom sequelae evolves throughout recovery; for example, distinct patterns of cerebral blood flow and white matter microstructure have been shown to relate to different symptoms at different time points post-injury, further illustrating the dynamic and ever-shifting relationships between network and experiential scales of concussion recovery (5, 59–61).

**Figure 2:**
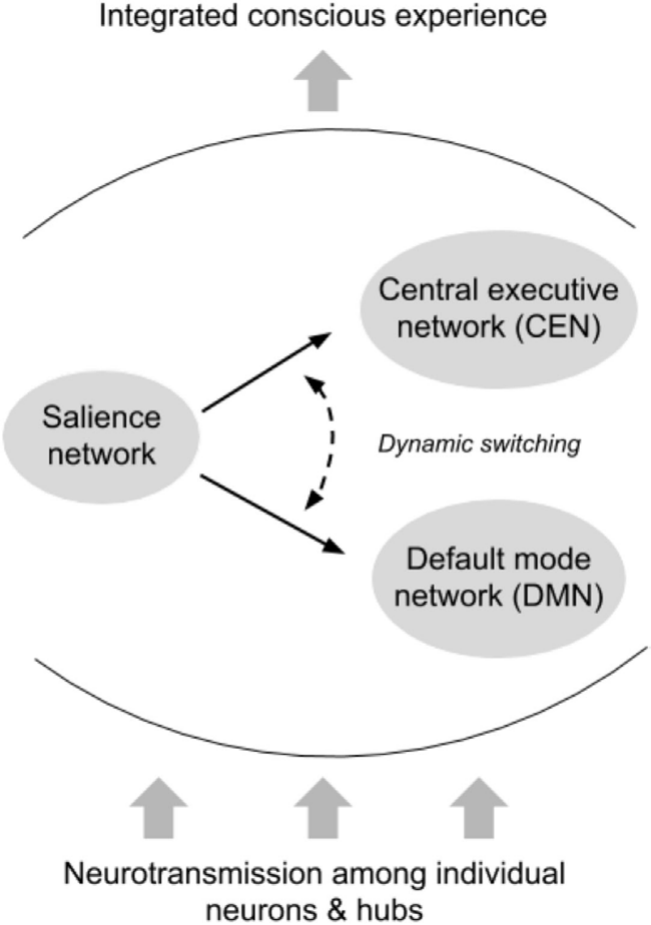
Interactions between intrinsic connectivity networks. The default-mode network (DMN) (64) is a highly coordinated network of hubs throughout the brain connected by long-range white matter tracts. The DMN is thought to be activated while an individual is at rest, and deactivated during goal-directed tasks (although there is some evidence that external tasks requiring social working memory may engage the DMN). In general, however, the DMN and other resting-state networks are deactivated once an individual begins task processing associated with external stimuli. Operating as a dynamic switch, the salience network deactivates the DMN and activates the central executive network, or *vice versa*. Successful switching between networks requires sensitivity to contextual demands, integration of multiple sources of information, and rapid appraisal, all of which are compromised with impaired or slowed neurotransmission. The dependence of such networks on long-range white matter tracts renders them particularly susceptible to the types of cellular insult observed in concussion.

Seamless interaction between networks in the connectome is necessary for integrated conscious experience. For example, the salience network provides the critical function of switching activation between the default-mode network and the central executive network (62, 63). This allows the individual to adjust between states of quiescence and rest and goal-directed tasks (see Figure 2).

Post-concussive symptoms often reflect network dysfunction not only within the primary network related to a given symptom expression but also *via* the primary network’s temporal or structural coordination with all other networks (65). Network correlates of single symptoms may shift and evolve throughout recovery, as will detection by the individual experiencing the symptom after sustaining a concussion.

#### Experiential

Network dysfunctions in concussion manifest in the individual as a variety of somatic, cognitive, and affective symptoms, such as mood disruptions, sleep disturbances, migraine/headache, impaired sensorimotor integration, and reduced cognitive processing speed (59, 64, 66–70). Abnormalities and asynchrony in signal processing result in cognitive fatigue, problems with sensorimotor integration, error signaling, and feeling out of sync (71). Sleep dysfunction as a post-concussion symptom is commonplace, which has a direct bearing on all brain networks because sleep is critical for glymphatic clearing of brain waste and provides a key feedback to the cellular scale by ameliorating local pathological microenvironments (72–74). Dysfunction in sleep patterns may lead to reduced cognitive load capacity and increased mood instability, and could interfere with social engagement—all problems experienced by patients with residual post-concussion symptoms (75).

Mood changes can originate in disruption of limbic and frontal networks and have social and behavioral effects (76–79). Psychological stress has wide-ranging consequences, from impaired brain-derived neutrophic factor (BDNF) expression and autonomic dysfunction to problems with sleep, mood, cognition, and coping (45, 80). Working memory and executive function have additional social and behavioral effects (81). Cognitive load capacity is affected by a variety of factors in the model, such as stress, exercise intolerance, need for cognitive rest, sleep, processing speed, and working memory, and executive function (82). Light or sound sensitivity can further exacerbate migraine headache, cognitive fatigue, and disorientation/confusion (83, 84). Problems with sensorimotor integration and vestibular function can cause issues with balance and gait, dizziness, and disruption in predictive timing of sensory input (82, 85). Disruption in the predictive brain state causes error signaling and its downstream effects (71).

#### Social

Cognitive and mood dysfunctions can impair an individual’s ability to function socially, which over time can erode the support they receive from strong personal relationships and integration in their communities (81). Strong social support and personal resilience are included in the model as positively impacting treatment, and coping and adaptation (86, 87). Social pressure, however, increases stress and interferes with adherence to treatment in the model. The model also includes several neuroprotective processes, such as cognitive rest, physical exercise, avoidance of overstimulation, and other treatment for headache, pain, and sleep dysfunction.

### Feedback

**Figure 3:**
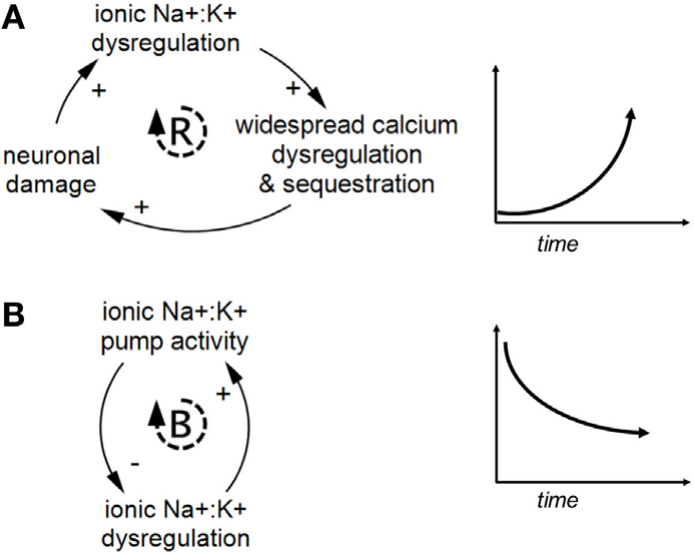
Examples of reinforcing and balancing feedback. Panel **(A)** shows a reinforcing feedback loop and a corresponding graph of exponentially increasing behavior over time. Panel **(B)** shows a balancing feedback loop and a corresponding graph of decreasing behavior over time toward an internal set point, based on ionic pump activity attenuating ionic dysregulation. Reinforcing and balancing feedback are the two types of feedback loops found in complex systems.

A key benefit to causal-loop diagramming is that it makes feedback relationships explicit. Feedback loops take two forms: reinforcing and balancing (see Figure 3). Reinforcing loops are “vicious” or “virtuous” cycles or cascades of exponential growth or decline. These loops can push the system out of balance in one direction or another when left unchecked. In contrast, balancing loops indicate repair, replenishing, homeostatic, or otherwise restorative processes and can be viewed as influencing progress toward a set point, goal, or neutral state.

**Figure 4:**
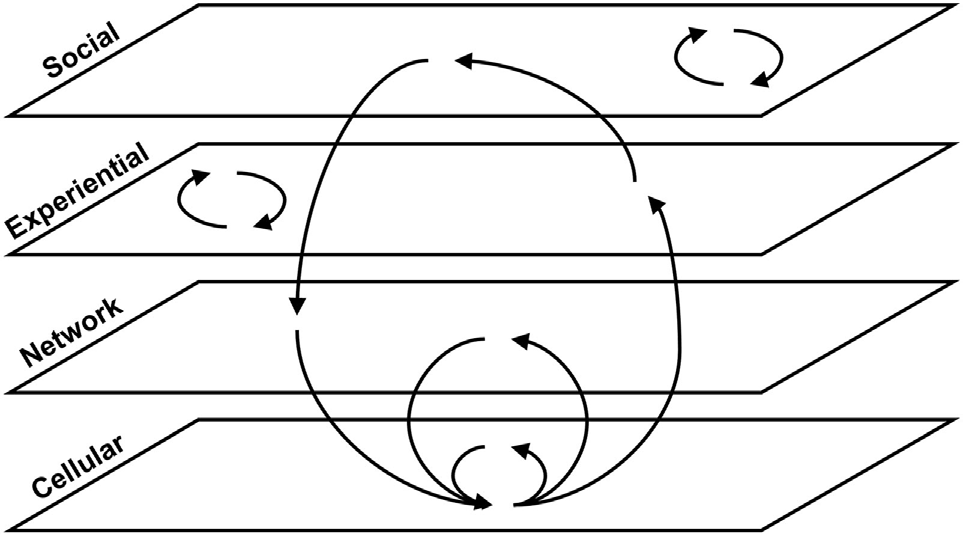
Within-scale and cross-scale feedback loops. Generic feedback loops are shown across four scales of organization relevant to concussion pathophysiology and recovery. Some loops occur within a given scale, and others span multiple scales.

Feedback loops exist both within specific biological scales and across multiple scales in the model. Figure 4 provides a generic illustration of feedback within and across scales in concussion. Upward arrows from smaller to larger scales can indicate emergence, as with individual neurons assembling into networks [see Ref. (19) for further discussion].

#### Within-Scale Loops

Short loops existing within one scale are denoted with R or B-loop icons in Figure 1. These loops indicate processes that often fall within the purview of a particular subfield of medicine or research discipline. At the cellular level, balancing loops for ionic pump activity, cellular repair, and immune response help to address damage and imbalance in the cellular milieu. Reinforcing loops related to glutamate release and ionic dysregulation show the cascading processes behind the neurometabolic cascade identified in concussion. A series of reinforcing loops around neuroinflammation show how inflammation can trigger processes that further exacerbate inflammation. A loop between the blood–brain barrier, pathological microenvironment, and neuroinflammation plays a particularly important role in prolonged recovery (46, 47, 88, 89).

**Figure 5:**
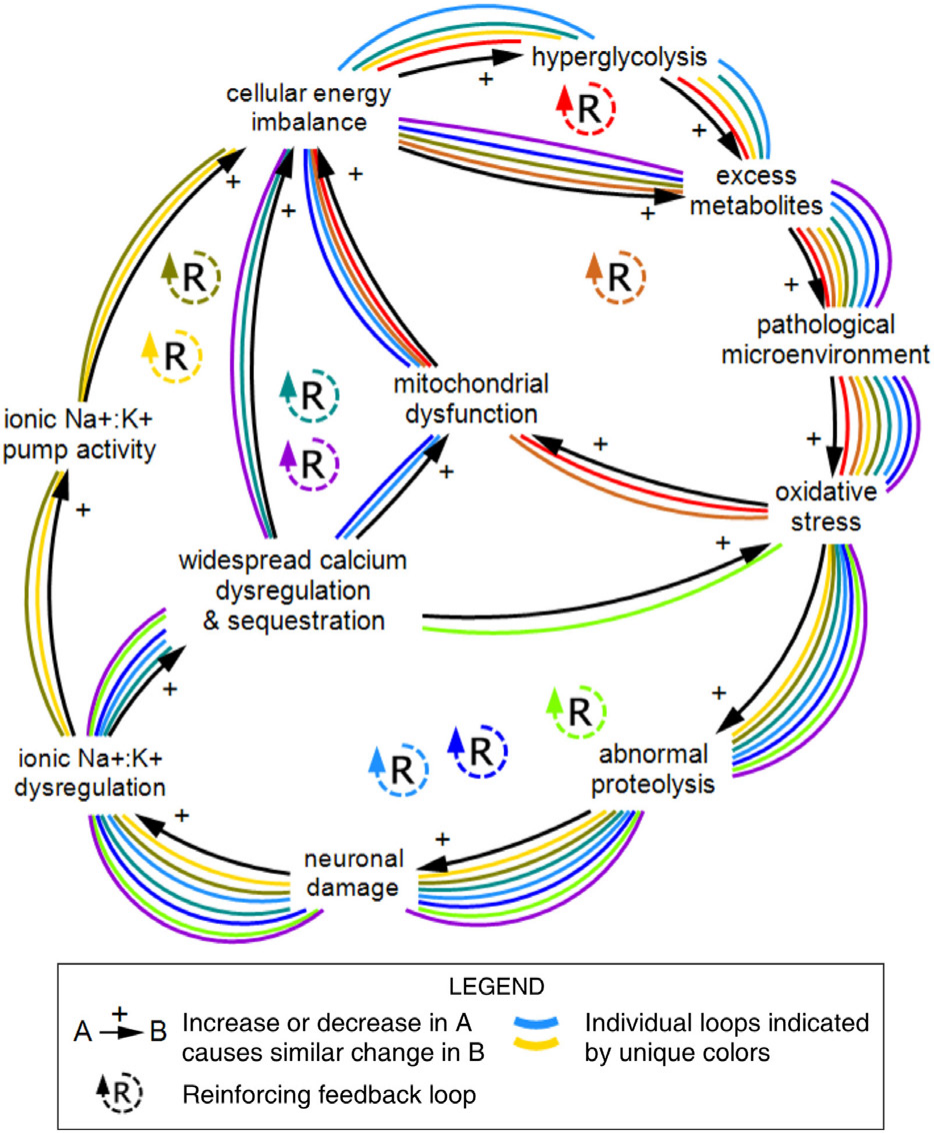
Nine feedback loops within the cellular scale of concussion. A series of reinforcing loops across the metabolic, ionic, and neuronal subsystems demonstrate the large number of feedback relationships that emerge from connections between a relatively small number of variables. The reinforcing structure of these relationships indicates compounding effects over time. Individual feedback loops are marked with unique colors. Diagram rendered in MapSys.

Larger within-scale loops not marked with loop icons can also be found in Figure 1. Figure 5 shows a series of nested reinforcing feedback loops within the cellular scale describing relationships between the metabolic, ionic, and neuronal subsystems. Individual feedback loops are indicated in this diagram to demonstrate the large number of loops that emerge from a relatively small number of variables and connections.

Within the network level, several short balancing loops describe how neuroplasticity, neuronal rerouting, and reorganization work to rectify impaired neurotransmission, particularly hub disruption. In neural networks, if the original primary pathways cannot come back online, then backup systems attempt to take over, depending on the redundancy and parallel connections within any given network (58, 90). Successful reorganization is determined in part by neural reserve (91).

**Figure 6:**
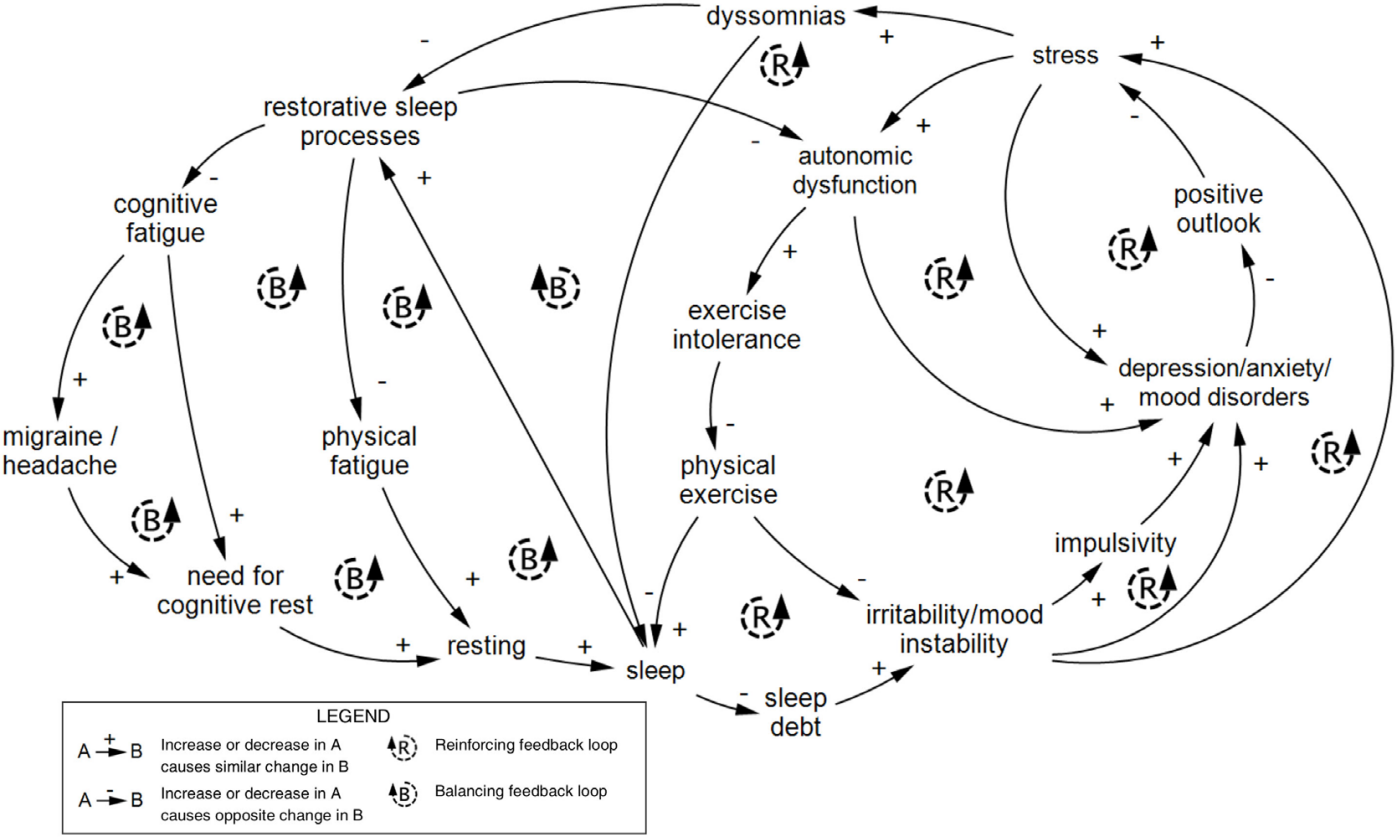
Feedback loops within the experiential scale of concussion. A series of nested feedback loops across the sleep/fatigue, autonomic, mood, and stress subsystems within the experiential scale are shown to illustrate the interconnectedness of variables across subsystems. This series of loops was reproduced from Figure 1. Diagram rendered in MapSys.

Sleep and cognitive fatigue feature a cluster of tightly connected balancing loops at the center of Figure 1. Figure 6 shows the interconnections between the sleep/fatigue, mood, stress, and autonomic subsystems. At the right side of Figure 1, these subsystems interlink with social variables, illustrating how emotional and social problems can compound one another. Figure 6 only depicts the connections described in Figure 1 and is not intended to comprehensively describe sleep dynamics.

**Figure 7:**
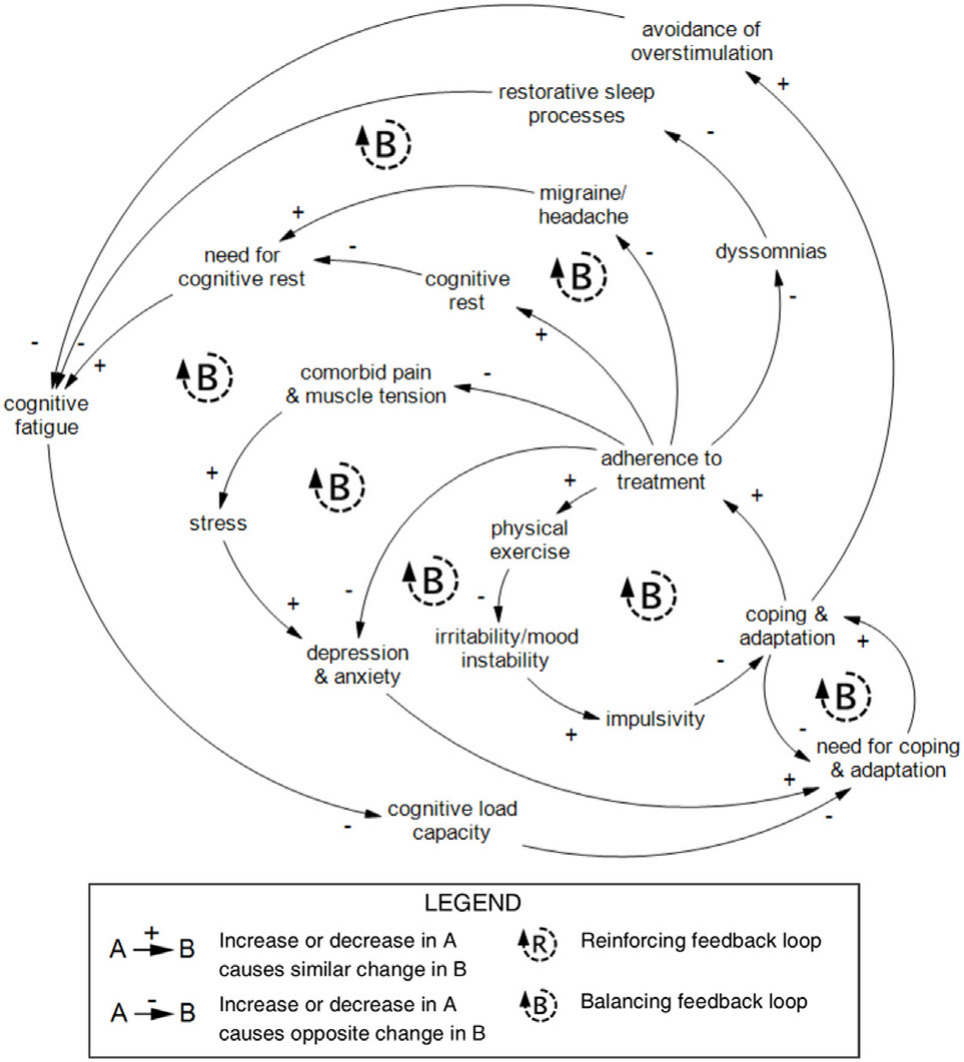
Balancing loops related to coping and adaptation within the experiential scale in recovery from concussion. This series of nested balancing feedback loops was reproduced from Figure 1. A core loop distinguishes coping and adaptation from the need for coping and adaptation. Coping and adaptation lead to two behaviors: avoidance of overstimulation and adherence to treatment. Avoidance of overstimulation reduces cognitive fatigue. Access and quality of medical treatment and advice will vary between cases, but targeted treatments may be prescribed by a clinician to increase cognitive rest or reduce migraine/headache, cognitive fatigue, comorbid pain and muscle tension, stress, depression and anxiety, irritability/mood instability, and impulsivity. Diagram rendered in MapSys.

A substantial number of loops within the experiential scale pertain to mechanisms of coping and adaptation that constitute balancing processes in the system. A series of multi-component balancing loops involving coping and adaptation on the right-hand side of Figure 1 are highlighted in Figure 7.

In several places in the model, a variable associated with a restorative process takes effect due to an increased need for repair or restoration, creating a balancing loop. For example, a need for cognitive rest results in more resting, which in turn decreases the need for rest. Distinguishing the *need for* something from the variable itself illustrates an endogenous goal-seeking process and makes distinct relationships with other subsystems explicit. Note that not all such processes have been specified in Figure 1.

#### Cross-Scale Loops

**Figure 8:**
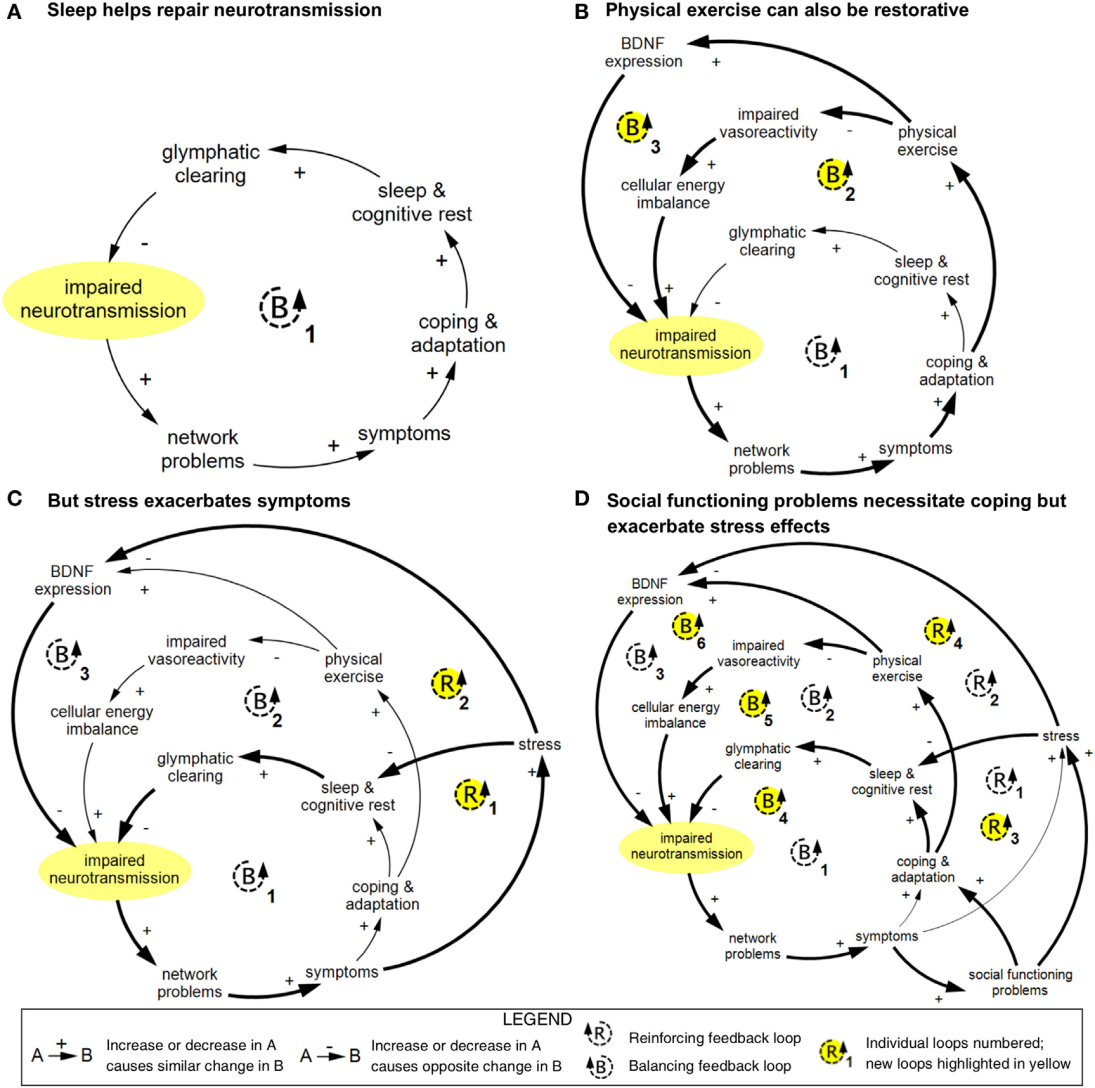
Simplified cross-scale feedback loops pertaining to impaired neurotransmission. These diagrams depict abbreviated versions of feedback loops described in Figure 1 and demonstrate how connected loops can have compounding and counteractive effects. **(A)** In loop B1, impaired neurotransmission affects the function of networks; these networks and network functions include limbic, intrinsic connectivity networks, attentional filtering, and processing speed. Disruption in these networks results in a range of symptoms included in Figure 1 (specifically, light or sound sensitivity, impairment in emotional regulation, impulsivity, irritability/mood instability, stress, depression/anxiety/mood disorders, reduced social functioning, impaired working memory and executive function, reduced cognitive load capacity, dizziness and vertigo, balance and gait problems, impaired prediction of sensory input, visual/perceptual impairment, disorientation and confusion, and reduced ability to work and complete daily tasks). Either directly or indirectly, these symptoms prompt coping and adaptation strategies, including avoidance of overstimulation, pursuit of and adherence to treatment, cognitive rest, and addressing of sleep problems. Restorative sleep processes lead to glymphatic clearing of brain waste and energy byproducts, which in turn results in improved neurotransmission *via* an improved cellular milieu and support of neuroplasticity. **(B)** In loop B2, physical exercise is used as a coping and adaptation strategy, which improves vasoreactivity and cellular energy imbalance, which supports neurotransmission. In loop B3, brain-derived neutrophic factor (BDNF) expression is strengthened, which reduces impaired neurotransmission *via* improved neuroplasticity. **(C)** Stress can disrupt sleep and inhibit BDNF expression, which creates two reinforcing loops. **(D)** Social functioning problems can prompt coping and adaptation, which introduces three additional balancing loops, and increase stress, which compounds the reinforcing effects of stress. Diagrams rendered in MapSys.

Including variables existing at multiple scales in one comprehensive model enables the identification of cross-scale feedback loops. Larger cross-scale feedback loops can be difficult to recognize, but can be critical to the structure of the system and, therefore, to how it operates. Many of the arrows in the concussion model (Figure 1) go from left to right, indicating emergence from smaller to larger scales (19). Several key arrows instead go from right to left, linking variables at the larger scale with variables at the smaller scale (indicated in bold in Figure 1). These “downscale” connections enable experiential and social processes to affect cellular and molecular processes, thereby creating a significant number of feedback loops in the model (summarized in Figure 8).

The connection between restorative sleep processes and glymphatic clearing in the concussion model (Figure 1) is particularly critical because it enables a series of balancing feedback loops pertaining to symptoms, network disruption, and impaired neurotransmission, the primary hub. Such loops feature a variety of neural networks and symptoms, but all tend to follow a similar basic structure (summarized in loop B1 in Figure 8A). Because sleep is a universal process, this loop provides the primary cross-scale restorative mechanism in the model.

Physical exercise also introduces balancing loops *via* its effect on expression of BDNF and impaired vasoreactivity (Figure 8B). An additional connection from a higher to lower scale is the link between stress and BDNF expression, which introduces several reinforcing loops in which stress exacerbates symptoms and slows recovery (Figure 8C). Problems with social functioning can prompt coping and adaptation, but also worsen stress and its effects (Figure 8D).

The downscale connections included in this model (pertaining to sleep, physical exercise, and stress) describe behaviors that have known biophysical components. As the psychological, emotional, and social dimensions of recovery are increasingly recognized (19, 81, 92), additional feedback relationships will be identified.

#### Loop Dominance

**Figure 9:**
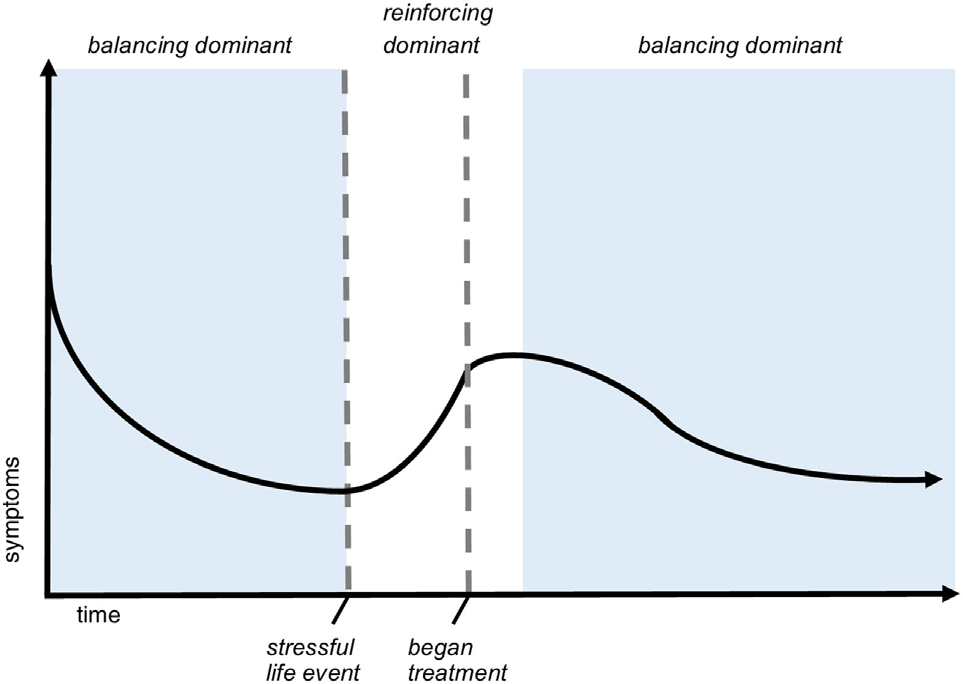
Shifting loop dominance evidenced in trajectory of symptoms over time. This hypothetical graph of symptom severity over time demonstrates a pattern of shifting dominance of interlinked feedback loops. In the scenario, the patient is injured at *t* = 0 and experiences a decreasing severity of symptoms. During this time (indicated by the first blue phase), balancing feedback is dominant. After a stressful life event, symptoms exponentially increase, meaning that reinforcing loops are dominant. Shortly after beginning treatment, balancing processes again dominate, indicated by the second blue phase. This sample recovery trajectory illustrates how feedback structure can cause nonlinear behavior of different types throughout recovery.

When loops interconnect, as shown in Figure 8, their relative influence can change over time, a phenomenon referred to as shifting loop dominance (35). For example, if the balancing loops in Figure 8 are dominant, impaired neurotransmission—and therefore symptoms—will be on a decreasing trajectory as the various coping and adaptation strategies take effect. But if the individual experiences a significant increase in stress due to either an increase in concussion symptoms or an unrelated stressor, for example, the reinforcing loops could become dominant, which would disrupt the patient’s recovery. Effective treatment could offset the effects of the stressful event and permit the balancing loops to once again dominate so that symptoms resume their downward trajectory. Figure 9 shows a sample recovery trajectory influenced by shifting loop dominance.

Examining loop structure and dominance within systems can facilitate greater understanding of nonlinear behavior of key variables of interest over time. It can also help explain how the system can get “stuck” in certain patterns, which in the case of concussion reflect the persistence of symptoms and deficits. For concussion recovery, which can be prolonged, unpredictable, and highly dependent on individual characteristics and context, this type of analysis is particularly suitable.

### Drivers

System drivers in a CLD are exogenous elements that affect variables in the system but are not themselves affected; they can be thought of as operating at the system boundary. Due to the large number of relationships already shown in the concussion CLD, only a few of the potential drivers are shown: impact, neural reserve, social pressure, and personal resilience. These elements represent aspects of the individual’s context and personal history that remain largely unchanged by variables endogenous to the system.

### Personal and Injury Characteristics

Concussion is highly heterogeneous, and myriad personal and injury characteristics shape recovery trajectories (21, 25). Several such characteristics were included in the concussion model (Figure 1) as drivers, as listed above. Although a comprehensive inventory of individual characteristics is outside the scope of this paper, presentation of the concussion model allows for the identification of several ways in which these characteristics interact with the system.

Some individual characteristics affect baseline levels of specific variables; for example, recent prior concussions could cause a degree of pre-injury hypometabolism in some individuals (42), while a history of migraines, depression, or sleep disruption would similarly alter baselines for those variables. Individuals with altered baseline levels could be more sensitive to certain feedback loops within the CLD, and as a result, be more or less prone to magnified symptoms following TBI. Also note that the non-specificity of symptoms of concussion complicates attribution in the absence of pre-injury baseline data.

Other characteristics could affect the efficiency or time course of certain processes, such as the immune response or glymphatic clearing, or affect the threshold at which symptoms occur. Certain characteristics, such as age and sex (85, 93, 94), have complex effects that significantly shape processes at all scales, both at the time of injury and dynamically through recovery.

Injury characteristics, such as amount of force (18, 95), rotational vs. linear acceleration (96), and injury context (83), also affect recovery. However, each human brain is uniquely wired. Individual differences and idiosyncrasies in neural connections and networks are present from the earliest stages of neurodevelopment and differentiate further with experience (97). Even if it were possible to determine *in vivo* how various biomechanical forces are translated from impact through the skull and layers of tissue into the human brain, it would still be difficult to make generalizations about the relationship between location of impact and function (23). At most, biomechanical studies have shown certain brain regions consistently experience some of the greatest strains and parenchymal deformations, particularly those in the subcortical white matter and its interface with cortical gray matter and the upper brainstem (18, 98). Outside of identifying areas generally more affected, the heterogeneity in modes of injury render most other claims of spatial localization ungeneralizable.

Many TBI studies include mixed etiologies of injuries and a wide-age range and do not control for educational and vocational backgrounds. All of these factors likely contribute unique influences at various levels in the systems models of brain injury, and none may impose a specific linear influence specifically related to outcome. Having a systems model provides a variety of options as to how to examine the multiple layers of influences at play in the evolution or resolution of symptoms following concussion.

### Boundaries

System boundaries are defined in by variables with no outgoing connections, as well as elements excluded from the diagram. In the present model, the “dead end” variables are loss of consciousness, vulnerability for future injury, risk for dementia and chronic traumatic encephalopathy, reduced reaction time, nausea, and feeling out of sync. Numerous elements and relationships pertaining to concussion were excluded from the diagram, including those that did not have a clear connection to predicting long-term outcomes, provided detail that was too granular, or were redundant of existing model structure. For example, monoamines are critical to the development and maintenance of emotion-related symptoms, such as depression, anxiety, mood disorders, and stress, but given that the disruption of limbic networks appear to be the primary levers and scales of interest for concussion recovery trajectories, this molecular-level detail was excluded from the model, as it would not have provided any new connections or information about the feedback dynamics driving symptom persistence and recovery. Significant variance or detail was also collapsed into certain summary variables. For example, specific social context dynamics about return to play or school can greatly affect certain individuals, but in our model, they are summarized in the exogenous variable *social pressure*. Additionally, the model describes impact concussion (caused by blunt force or accelerative/decelerative forces), and excludes blast-type concussions and other injury modalities.

### Hubs

In a network, hubs are nodes that are disproportionately connected with other nodes in the system (90, 99). The primary hub in the concussion CLD is impaired neurotransmission, which serves as a main conduit between the micro-scale left side of the diagram and the larger scales of symptoms and experience on the right side. The next most connected hubs in the diagram are stress, pathological microenvironment, and cognitive fatigue.

The concept of hubs is also important for understanding the structure of neural networks. As outlined by Bassett and Sporns (100), network neuroscience has demonstrated nonrandom topological attributes that relate to function, where neuroimaging has demonstrated “high clustering and short path length, and network communities (modules) linked by highly connected hub nodes that are in turn densely linked, forming an integrative core or rich club (p. 6).” In this context, damage to hubs can disrupt entire networks.

### Interventions

A comprehensive inventory of interventions for TBI is outside the scope of this model. However, several variables for coping, adaptation, and treatment were included in the concussion CLD (Figure 1) to demonstrate the possible role of intervention processes in recovery. For example, adherence to treatment helps to reduce dyssomnias, migraine, and headache, and comorbid pain and muscle tension, as well as to increase physical exercise and cognitive rest (83). Another coping mechanism is avoidance of overstimulation, which reduces cognitive fatigue in the model. Specific treatments, such as pharmaceuticals for migraine or therapy for visual/perceptual impairment, could be customized to the individual and might be seen as exogenous drivers to the system. Future precision medicine interventions targeting subgroups of patients based on genetic profile or other aspects of physiology could introduce additional feedback loops at certain points in the model.

In a heterogeneous system, interventions should respond to individualized needs based on physical, emotional, cognitive, and social dynamics. A truism in systems science is that obvious solutions often backfire; truly effective interventions must be sensitive to how interrelationships between variables play out over time. For example, balancing loops can be introduced, reinforcing processes can be tempered, and drivers can be addressed. If knowledge about concussion pathophysiology becomes sophisticated enough to be able to generate a CLD specific to an individual person, customized interventions could be identified based on an analysis of system structure, including loops and drivers.

## Discussion

Systems models such as the one presented here provide a way for different types of knowledge from multiple subfields to be integrated into a larger working hypothesis. For concussion, the heterogeneity of the phenomenon and the diversity of methods and measures for studying it support the need for a common understanding of basic system structure. Having such a “map” of the “terrain” of knowledge about a system can be useful in both research and clinical settings.

### Research Applications

As a reflection of current scientific knowledge, a systems model can facilitate the identification of research gaps and opportunities for interdisciplinary collaboration. One key observation made while building this model was the relative lack of research on restorative or ameliorative processes—particularly endogenous ones—in favor of pathological processes. Basic research into the body’s diverse healing mechanisms, as well as intraindividual variability of these mechanisms, would support a more comprehensive understanding of how symptoms lessen over time and perhaps lead to new treatments (101). Analysis of the feedback structure of this model also revealed the importance of connections from the larger to smaller scales in introducing feedback loops, particularly cross-scale balancing loops that reduce symptoms. Greater understanding of cross-scale loops and other similar downscale connections would be particularly useful for improving knowledge about recovery.

A model that brings together knowledge from a diverse range of subdisciplines provides a unique opportunity to identify key components and understand the magnitude of their influence on overall clinical presentation. This framework could serve as a platform for integration of research findings regarding new diagnostic markers (e.g., blood biomarkers) or pharmaceutical treatments. Such insight about how different types of evidence contribute to a larger understanding of concussion could inform efforts to develop a new classification system for TBI. To provide the most useful input for reclassification, systems models would be developed using high-quality time-course data from a wide variety of patients as well as robust studies documenting identified pathophysiological mechanisms. Although such data are not currently available, a preliminary hypothesis model could lay the foundation for future work by encouraging such research and providing a platform for integration of different types of data.

Such a platform could be used in an iterative fashion with other research strategies such as traditional basic and clinical research and systematic reviews to work toward shared goals such as the development of clinical care guidelines. Systems approaches also serve particularly well as a complement to big-data approaches, which generate insight in a bottom-up manner directly from data, as well as personalized systems biology methods that use individual-scale “-omic” data to predict risk and recovery (102–104). When used in conjunction with these other approaches, visual systems methods such as causal-loop diagramming can serve as a valuable platform for interdisciplinary discussion, hypothesis generation and theory building, and spur methodological innovation.

Causal-loop diagrams, which depict hypothesized causal relationships between aggregate quantities, also complement current applications of network neuroscience and graph theory relating brain to neurobehavioral functioning (90). Advanced neuroimaging methods that track how networks respond to injury may ultimately prove useful in guiding treatment and outcome (105), although initial efforts have been disappointing in sports concussion (106). Systems methods might bring to this analysis useful attention to functional dynamics over time.

### Clinical Applications

A systems approach can inform clinical decision-making both indirectly through the outputs of research mentioned above (i.e., clinical care guidelines, diagnostic measures, and treatment protocols) and directly by providing clinicians with a new way of viewing and analyzing concussion. As with most of medicine, clinical diagnosis, prognosis, and treatment of concussion involves pattern recognition. This model could help clinicians visualize patterns that are currently hidden in the complexity of the clinical scenario in order to better identify those individuals at high risk for poor recovery and link treatments to appropriate outcomes and measures.

Awareness of feedback mechanisms is particularly relevant in the clinic. Figure 1 illustrates how balancing processes related to sleep, cognitive rest, physical exercise, coping and adaptation, neuroplasticity, and cellular repair contribute to symptom reduction. When identifying treatment plans for concussion patients, clinicians could consider how balancing processes (e.g., sleep, cognitive rest, physical exercise, coping and adaptation, neuroplasticity, and cellular repair) might be enhanced, and how reinforcing processes (e.g., stress and social dysfunction) might be mitigated.

Another clinical takeaway is that factors endogenous to the system are more likely to persist over time. Prominent systems scientist Donella Meadows identified 12 ways to intervene in a system, organized by increasing capacity for transformative change (107). Within her framework, reorganization of system structure often has greater leverage than minor changes to variables, especially temporary or exogenously driven changes. In the clinic, this could mean helping patients to identify sustainable changes to habits, lifestyle choices, and social support that could complement and strengthen more traditional, exogenously driven therapies and treatments. For example, proper sleep hygiene and stress reduction can reduce sleep disruption, and avoidance of overstimulation and certain triggers can reduce cognitive fatigue and migraine. Endogenous means of behavioral adaptation shape the system in ongoing ways, while clinic-centered treatment can be time limited. Attention to behavioral adaptation in the clinic is established practice, but the causal-loop diagram provides a new rationale for this strategy.

### Modeling Challenges

Modeling complex systems inevitably involves a tradeoff between comprehensiveness and legibility. The concussion model presented in Figure 1 is a qualitative representation of the modelers’ understanding of the system at a certain point in time. It is, therefore, defined by the main data and themes identified during the course of research and does not include every aspect of concussion recovery.

Ideally, systems models would be living documents continually updated according to the latest medical knowledge and applied as heuristic tools in an iterative fashion. A static, two-dimensional image is also not the ideal presentation for a model with so many possible layers of information. The web-based version of the model allows users more control in navigating the model and viewing supporting information.

Model development was complicated by a lack of clarity in the literature regarding the extent to which research on TBI, broadly defined, also applies to mTBI or concussion. Whether injuries of different severities and of different types indeed belong on a single continuum is a matter of controversy (108). More precision and consistency in TBI classification, definition, and measurement would provide the basis for more clarity in findings from basic and clinical research.

The non-specificity of symptoms and the effects of litigation complicate TBI research and, therefore, model building. As outlined in recent reviews to understand vulnerabilities related to concussion outcome, consideration must include a host of both pre- as well as post-injury factors (109, 110). Individuals with prior neuropsychiatric conditions, especially depression, anxiety, and pain-related disorders, are particularly vulnerable to developing residual problems after a concussive brain injury. Frontal–temporal–limbic systems play a role in neuropsychiatric symptomology and are also likely injured in concussion. Neuropsychiatric symptoms also relate to symptom reporting, including symptom magnification following injury (111). Presence of litigation represents a significant issue in TBI outcome studies (112). Hiploylee and colleagues (5) demonstrate how a comprehensive approach to symptom reporting and validity testing using a longitudinal design can help control for such effects. If such methods become more commonplace, future reviews and models might preference them to identify a more precise picture of the effects of concussion.

Causal-loop diagramming does not readily depict changes in system structure over time. Both cognitive rest and physical exercise, for example, have been shown to be beneficial or harmful depending on the amount of time following injury (113). Because in-depth modeling of this subsystem was outside the scope of this project, the concussion model in Figure 1 includes only the benefits of rest and exercise. Spatial localization of injury, particularly regarding neurological damage and network disruption, is also difficult to represent with a diagram methodology structured around aggregate quantities.

Many of the challenges faced in modeling concussion—such as classification uncertainty, heterogeneity, and diversity of measures—reflect challenges stemming from the complexity of concussion itself that are present, but not always fully acknowledged, in traditional TBI research. For example, the failure of promising Phase III clinical trials for TBI treatments may say more about the lack of precision in the definition and measurement of TBI than the potential effectiveness of the proposed treatments for particular subgroups (114).

### Future Work

The model described in this article is a demonstration of an innovative methodology and serves as a proof of concept for future systems-oriented efforts to understand TBI. This model could be further developed to include a more detailed depiction of certain subsystems, particularly at the network, experiential, and social scales. As scientific knowledge about concussion expands, the model could be adapted to reflect the changing consensus. Such a “living model” would more accurately depict the current state of knowledge about concussion and would, therefore, provide a more timely basis for clinical and research applications. The web-based version of Figure 1, which enables public commenting on specific model elements and relationships, is a step in this direction. However, procedures and resources for updating such a model on an ongoing basis have not yet been determined.

Future modeling efforts could include the development of computational system dynamics models based on the concussion CLD. System dynamics models are a logical extension of CLDs and introduce the dimension of time to the model, which allows for a more sophisticated examination of recovery trajectories and leverage points based on the operationalization of variables. Feedback mechanisms and the influence of personal and injury characteristics could, therefore, be analyzed in more detail. Specification and testing of these models, however, requires time-course data of key system variables, which is currently lacking in TBI research.

Specific hypotheses identified using this model could also be tested using other methods. For example, research to test the hypothesis that downscale connections have outsize influence on system behavior could be conducted either experimentally or using a computational model. Neuroimaging-based neural networks could also be derived that correspond to some of the hypothetical networks depicted in Figure 1.

## Conclusion

This research has shown that applying systems methods to concussion yields insight that is applicable in both research and clinical settings. Identifying key loops and drivers and considering how loop dominance may shift, either from endogenous or exogenous factors, is crucial to understanding the ways that post-concussive symptoms persist or resolve over time. By using systems modeling in conjunction with other new and more traditional approaches, a potentially fruitful new area of research could provide the synthesis and analysis necessary to address the heterogeneity and complexity found in concussion—a crucial step toward improving clinical outcomes.

## Author Contributions

EK was the lead modeler and lead author. EP and WW contributed substantially to the model and article. EK, EP, and WW conducted expert interviews and literature review. EB, DW, ML, JC, GH, and WG provided critical review and revision of the model and article. WW also provided systems modeling guidance.

## Conflict of Interest Statement

The authors declare that the research was conducted in the absence of any commercial or financial relationships that could be construed as a potential conflict of interest.

## Footnotes

^1^Here, we choose to use these terms interchangeably, but recognize the ongoing debate in the literature (1, 2). Although *concussion* is arguably more colloquial than *mTBI*, it is flexible enough to accommodate the development of a new classification system (whereas mTBI is tied to the Glasgow Coma Scale). We do intend to be inclusive of complicated mild cases with prolonged recoveries.

^2^https://www.commondataelements.ninds.nih.gov/ (Accessed: March 17, 2018).

^3^https://fitbir.nih.gov/ (Accessed: March 17, 2018).
