# Supplementary material for: The Dynamics of Concussion: Mapping Pathophysiology, Persistence, and Recovery With Causal-Loop Diagramming
Source: Front Neurol. 2018 Apr 4;9:203. doi: 10.3389/fneur.2018.00203 (PMC5893805; doi:10.3389/fneur.2018.00203)
Supplement: Supplementary file 1 [file Table_1.PDF]

Interviewee List

Semi-structured interviews were conducted with the following individuals during the early phases of model development:

P. David Adelson, MD
Barrow Neurological Institute at
Phoenix Children's Hospital

David Arciniegas, MD
Professor of Psychiatry, Neurology, and Physical Medicine & Rehabilitation
Baylor College of Medicine

Erin Bigler, PhD
Department of Psychology and Neuroscience Center
Brigham Young University

James Chesnutt, MD
TBI/Concussion Program, Orthopedics & Rehabilitation and Family Medicine
Oregon Health & Science University

Robert Clark, MD
Chief, Pediatric Critical Care Medicine
University of Pittsburgh School of Medicine

Michael W. Collins, PhD
Director, UPMC Sports Medicine Concussion Program
University of Pittsburgh School of Medicine

Jamshid Ghajar, MD, PhD
Director, Concussion and Brain Performance Center
Stanford University

Wayne Gordon, PhD
Professor, Department of Rehabilitation Medicine
Icahn School of Medicine at Mount Sinai

Gregory Hawryluk, MD, PhD
Department of Neurosurgery
University of Utah

David Hovda, PhD
Director, UCLA Brain Injury Research Center
Professor, Departments of Neurosurgery and Molecular and Medical Pharmacology
David Geffen School of Medicine at UCLA

Jeffrey J. Iliff, PhD
Associate Professor, Anesthesiology and Perioperative Medicine
Oregon Health & Science University

John Leddy, MD
Clinical Professor, Primary Care Sports Medicine, Dir. UB Concussion Management Clinic,
University at Buffalo, SUNY

Miranda Lim, MD, PhD
Sleep Disorders Clinic, Division of Hospital and Specialty Medicine
Veterans Affairs Portland Health Care System
Departments of Neurology, Medicine, and Behavioral Neuroscience, and Oregon Institute of Occupational Health Sciences
Oregon Health & Science University

Thomas McAllister, MD
Chairman, Department of Psychiatry
Indiana University

Gregory Petsko, D. Phil
Arthur J. Mahon Professor of Neurology and Neuroscience and Director, Helen and Robert Appel Alzheimer's Disease Research Institute
Feil Family Brain and Mind Research Institute, Weill Cornell Medical College
Professor of Biomedical Engineering, Cornell University

Jennie Ponsford
Professor of Neuropsychology, School of Psychological Sciences, Monash University
Director, Monash-Epworth Rehabilitation Research Centre, Epworth Healthcare, Melbourne, Australia

John Povlishock, PhD
Professor Department Head, Department of Anatomy and Neurobiology
Virginia Commonwealth University School of Medicine

Eric Schnell, MD, PhD
Assistant Professor, Anesthesiology and Perioperative Medicine
Oregon Health & Science University

Doug Smith, MD
Department of Neurosurgery
Perelman School of Medicine
University of Pennsylvania

Bob Stern, PhD
Professor of Neurology, Neurosurgery; and Anatomy & Neurobiology
Boston University

Mark Wainwright, MD, PhD
Professor of Pediatric Neurology, Neurology, and Pharmacology
Feinberg School of Medicine
Northwestern University

Alan H Weintraub MD
Medical Director, Brain Injury Program Craig Hospital
Rocky Mountain Regional Brain Injury System
Associate Clinical Professor, University of Colorado School of Medicine

David Wright, MD
Department of Emergency Medicine
Emory University School of Medicine

Mariusz Ziejewski, PhD
Professor, Engineering, Dir. Impact Biomechanics Laboratory
North Dakota State University

Nancy Carney, PhD and Hugo du Coudray, PhD contributed substantively to early phases of model development but were not formally interviewed.
